# Collicular circuits supporting the perceptual, motor and cognitive demands of ethological environments

**DOI:** 10.1016/j.conb.2023.102773

**Published:** 2023-08-22

**Authors:** Daniel de Malmazet, Marco Tripodi

**Affiliations:** MRC Laboratory of Molecular Biology, Cambridge, UK

**Keywords:** Superior colliculus, Tectum, Ethology, Sensory-motor processing, Prey capture, Cognition, Region-specific anatomical and functional properties, Flexible sensorimotor transformations, Neural circuits

## Abstract

Animals evolve to survive in their environment. Accordingly, a reasonable hypothesis is that brain evolution prioritises the processing of useful sensory information over complete representation of the surroundings. The superior colliculus or tectum is a brain area that processes the animal’s surroundings and directs movements in space. Here, we review recent studies on the role of the superior colliculus to assess the validity of this “utility hypothesis”. We discuss how the response properties of collicular neurons vary across anatomical regions to capture ethologically relevant stimuli at a given portion of the sensory field. Next, we focus on the recent advances dissecting the role of defined types of sensory and motor neurons of the colliculus in prey capture. Finally, we discuss the recent literature describing how this ancient structure, with neural circuits over 500 million years old, implements the necessary degree of cognitive control for flexible sensorimotor transformation.

## Introduction

The superior colliculus (SC) is a midbrain structure essential for controlling sensory driven spatially oriented actions. Broadly speaking, the SC contains two major domains dominated respectively by sensory and motor responses. The sensory domain maps the surrounding space using visual input alongside other modalities such as auditory input, electroreception, vibrissae or even infrared depending on the species [1]. While much of the past literature has focused on the sensory and motor domains separately, we argue here that the SC offers an ideal system to pursue an integrative approach for the study of circuit, molecular and computational underpinnings of flexible sensory-motor loops supporting ethologically relevant behaviours. We also argue that the flexibility of these sensory-motor integration strategies fulfils many of the characteristics of cognitive control, hence providing a tractable system to study circuit and molecular mechanisms underpinning cognitive functions. We focus in this review on the most recent literature concerning the role of the SC in flexible sensory-motor control in rodents, fish and monkeys and highlight the strengths of an integrative and ethological approach for the study of sensorimotor integration and cognitive control.

## Perception. Seeing what matters, where it matters. How the intrinsic organization of the superior colliculus prioritises ethologically relevant stimuli

One of the best recognised anatomical-functional principle in the SC is the ordered retinotopic organization of receptive field responses in its superficial layers. Namely, the upper-lower visual field axis is mapped along the medial-lateral SC anatomical axis and the posterior-anterior visual field axis is mapped onto the caudal-rostral SC. This straightforward organisational principle has led to the idea that the SC is a transparent sensory station acting as a camera onto the world, faithfully and orderly representing the external visual scene.

However, recent findings revealed a more nuanced and less transparent collicular encoding of the sensory world, casting doubts on a simplistic “camera view”. Notably, higher-level visual response properties, such as orientation and direction preferences, show prominent region-specific biases [2–9]. Specifically, cells preferring the same orientation and/or movement direction tend to cluster together, forming large direction/orientation columns that span a diameter of 100–200 μm, equating to a field of view of roughly 30°. These newly observed columnar clustering would therefore lead to biases in how the visual field is processed. Namely, in addition to the increased sensitivity of collicular neurons for moving stimuli [10], these new findings indicate that collicular neurons prefer stimuli with a specific direction or orientation depending on the stimulated region of the visual field.

**Figure 1:**
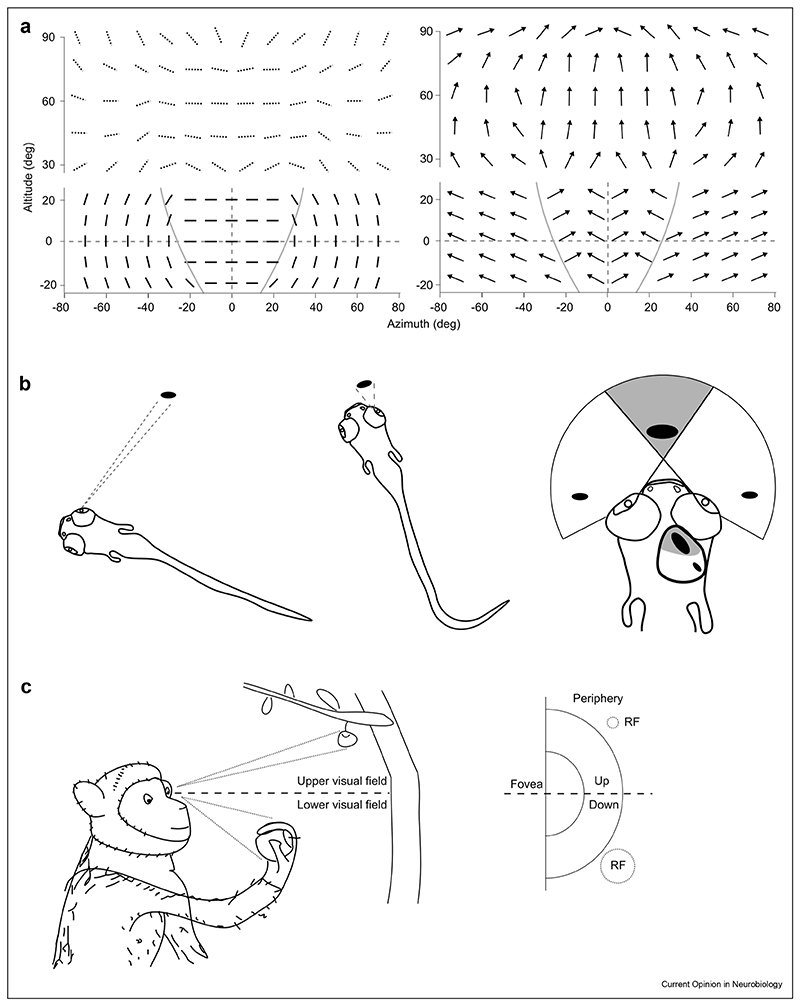
Region specific maps of visual responses in the SC of mice, zebrafishes and monkeys. **a**. Visual field representation of orientation and motion direction preferences in the SC of mice. In the left panels, the preferred orientation at each visual field location is represented by a bar orientation. In the right panels, each arrow represents the preferred direction of motion encoded at each visual field location. The bottom panels indicate the preference at around ground level. The top panels show the response preferences in the upper visual field. The two grey curves in the bottom panel represent the border between the monocular and binocular zones. 0-degree altitude corresponds to the elevation of the Bregma-Lamda axis and 0-degree azimuth corresponds to plane passing by the nose and in-between both eyes. The bars in dotted lines in the orientation panel represent the inferred preferred orientations based on the orthogonal relationship with the preferred motion direction in the upper visual field [5]. **b**. Preference for larger dots in the rostral part of the zebrafish’s tectum compared to the caudal part. Left drawings illustrate the visual angle of preys when far or close to the fish. Right drawing shows in grey the binocular zone of the zebrafish. The difference in size preference of visual stimuli between the rostral and caudal parts are depicted both in the tectum and in the visual field. **c**. Larger receptive fields sizes for cells in the SC sampling the lower part of the visual field in monkeys. Left drawing illustrates the differences in visual angle when the same object is far or close to the eyes of the monkeys. The lower part of the visual field often includes objects held in hand and therefore closer to the eyes, while objects in the upper part of the visual field are usually located far from the monkey. Drawing in the right illustrates the difference in receptive field size found in the monkey SC for cells sampling the lower versus upper portions of the visual field.

When assigning the direction and orientation preferences of each individual cluster to a global map of response properties across the visual field, the potential ethological relevance of this organisation begins to emerge (Figure 1a). The orientation preferred at each point is tangential to a circle centred around the animal’s nose, with cells sensing the binocular zone preferring horizontal orientations [7,8]. Furthermore, direction-selective cells are tuned mainly to upward directions throughout the visual field, but their precise direction selectiveness depends on their retinotopic location [5,7]. Specifically, in the upper visual field, their preferred direction aligns with the optic flow formed onto the retina as if the mouse would fall vertically or an object would approach from above [5]. For the visual field at the ground level, direction-selective cells sensing the binocular zone prefer objects moving towards the nose, whereas those sensing the monocular zone respond preferentially to stimuli moving away from the nose [7].

In other words, rather than faithfully representing stimuli as they are in the external world, the SC appears to prioritise specific features depending on where the stimulus appears in the visual field in a manner that reflects the statistics of natural visual scenes. Moreover, when reflecting on the nature of the preferred responses at each visual field location there is a sense that these responses tend to prioritise ecologically relevant kinetic features for the overseen portion of the visual scene. For example, the prioritization of downwards directed approaching stimuli in the upper visual field, would appear to fulfil the ethological need to identify and avoid approaching predators. On the other hand, the prioritization of horizontal moving object in the binocular zone of the lower visual field, would seem to satisfy the ethological need to detect and intercept preys [11,12], whereas the higher sensitivity to vertical orientations and motion directed away from the nose in the monocular zones would create a propension to detect the optic flow resulting from self-generated movement and serve a function in orienting [12].

While some debate is still ongoing on the precise nature and general relevance of these organizational principles [4,13], the existence of regional prioritization strategies for ethological relevant stimuli is reinforced by studies in other species.

For example, in fish, the typical hunting cycle is composed by a detection phase of the potential prey in the peripheral field of view, in which the prospective prey would typically cover no more than 5° in visual angle. Such a phase would typically be followed by reorientation and approach in such a way that the prey would enter the central binocular zone and expand to cover around 30° of the visual field [14]. Coherently with the ethological need of a successful hunting cycle, neurons in the caudal part of the optic tectum, which cover the peripheral field of view, tend to prefer smaller stimuli than neurons located in the rostral region of the tectum, covering the central field of view [9] (Figure 1b). Hence, also in the fish, instead of a homogenous faithful and location-independent mapping of visual properties, we observe a receptive-field dependent prioritization of visual features that matches the underlying statistics of natural scenes and that favours the execution of purposeful ethological behaviours, such as hunting pursuit.

Similarly to what was observed in fish and mice, studies in primates also point towards a significant functional regionalization in the SC. Specifically, in the case of primates, the lower and upper visual fields tend to carry very different types of information in ethological conditions, with the lower visual field normally conveying information about near space and the upper visual field typically conveying information about distant small objects (Figure 1c). Recent work shows how the primate SC organization reflects these ecological constraints with more accurate and lower-latency saccades towards upper visual field objects than lower visual field targets and an overall anatomical over-representation of the upper visual field. Coherently, the study also shows that receptive fields in the upper visual field are smaller than those in the lower visual field, leading to a perceptual magnification and a better resolution of the upper visual field similar in principle to the better-known foveal magnification [2].

Overall, when looking at these new findings regarding stimulus representation in the SC, two key evolutionary conserved organizational principles seem to emerge. The first is that it exists an interdependence between receptive field location and higher visual features representation that reflects the statistics of natural environments. An example of this first principle can be seen in the case of the upper visual field magnification that has been described in primates and that would seem to reflect the overwhelming presence of distant small objects in the upper visual scene in natural environments [2]. The second principle that emerges is that visual feature responses that align better with the ethological needs of the animal tend to be prioritised by evolution, granted that they respect the first constraint of a meaningful representation of natural scene statistics. A point in case for this second principle can be seen in the prioritization of horizontal motion in the lower visual field in rodents that appears to favour hunting behaviour [7].

The highly stereotypical response to looming stimuli represents another fine example of a behavioural response mediated by the SC that is conserved across all vertebrates and that satisfies both principles enunciated above [15]. In mice, an overhead fast expanding black disk elicits strong defensive reactions, mimicking the natural defensive behaviour triggered by an approaching aerial predator [16]. The medial region of the SC, which senses the upper visual field, responds strongly to overhead stimuli [10] and initiate an escape or freezing response depending on the kinetic features of the stimulus.

## Action. Intracollicular circuit motives underpinning sensorimotor integration for key ethological behaviours

As we observed when discussing stimulus prioritization in the fish tectum, the study of predation provides fertile ground to dissect the organizational principles underlying sensory motor transformation in the SC. This is supported by a plethora of recent studies focusing on how the superior colliculus or optic tectum control ethological behaviours both in mice and in fish [17–24]. Since recent reviews have already amply and competently discussed the role of the SC in predatory evasion and escape [16], here we will focus specifically on recent advances regarding the role of the SC in predation. In particular, we will focus on the role of specific collicular cell types supporting the sensory motor transformation necessary for the different phases of the execution of prey capture (Table 1).

A clear evidence of the involvement of the SC in hunting behaviour in rodents comes from its bilateral inactivation showing reduction in the hunting performance of mice [17]. To further dissect the function of the SC in predation, several recent studies tested the role of specific collicular cell types. From a sensory perspective, the response properties of neurons in the superficial layers of the SC have been characterised in some details. Broadly speaking, four non-overlapping sets of visual responsive cells can be identified based on the width of their receptive filed responses and dendritic morphology: horizontal cells, stellate cells, wide field and narrow field cells [25]. With regard to the latter two types, wide field cells are characterised by large dendritic arbors, respond to stimuli within a large region of the visual field and prefer small kinetic stimuli. Narrow field cells, as implied by the name, have small receptive fields and narrow dendritic arbors, also prefer kinetic stimuli and show strong direction selectivity for moving stimuli. Beside their characteristic response properties, these cells are also molecularly defined and labelled using available CRE mouse lines. Narrow field neurons are characterised by the selective expression of the Gastrin Releasing Peptide (Grp) and are labelled by the Grp-KH288 line. Wide field neurons are well labelled by the Ntsr1-GN209 line albeit their expression of the Neurotensin Receptor 1 gene (Ntsr1) remains unclear [25,26]. This enables a detailed characterization of the role of these two populations in behaviour.

One study in particular reported opposite effect on hunting when inactivating these two cell types from the superficial layers of the SC. Hoy and colleagues [18] inhibited Ntsr1^ON^ wide field neurons and found that it prevented mice from initiating approach towards the prey from afar. However, it did not affect the mouse’s ability to intercept and pursue the prey when in close range. On the contrary, when inhibiting GRP^ON^ narrow field neurons, they saw no effect on the initiation of movement towards the prey, but instead reported that mice’s ability to pursue and attack the prey was impaired [18].

Interestingly, inactivating parvalbumin or cerebellin 2 precursor (CLBN2) positive neurons in the superficial layers of the SC does not affect hunting [18,23]. This is surprising because parvalbumin and CLBN2 positive neurons overlap with Ntsr1^ON^ wide field neurons in the stratum opticum and all these neurons project to the lateral pulvinar nucleus of the thalamus [18,23]. In addition, a recent study [23] showed that nearly 90% of collicular neurons projecting to the lateral pulvinar are CLBN2 positive. This suggests that only specific cell types are implicated in hunting and that parallel circuits sharing similar connectivity features can oversee different behaviours.

**Figure 2:**
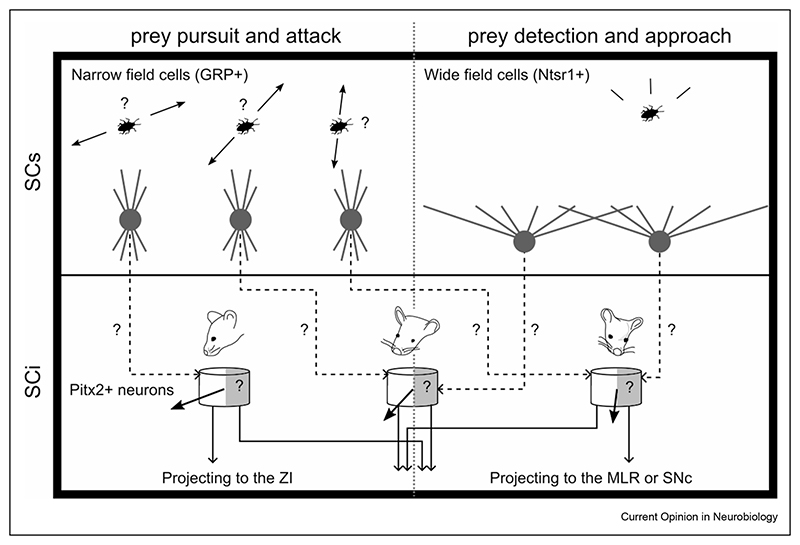
Illustration of putative parallel collicular circuits and sensorimotor mechanisms supporting prey attack and approach. Narrow field cells in the SCs were shown to exhibit direction selective properties. Given they share the same involvement as Pitx2^ON^ in prey pursuit and attack, we hypothesize they could provide input to Pitx2^ON^ in the intermediate layers. With that regard, it would be interesting to understand whether GRP^ON^ and Pitx2^ON^neurons share any spatial preference with respect to either visuo-motor direction selectiveness or visuo-motor receptive fields. Similarly, because wide field neurons in the SCs and MLR and SNc projecting neurons in the intermediate and deep layer are involved in the initiation of prey approach, we hypothesize that these cells might be anatomically connected. Given that Pitx2^ON^ neurons also project to the MLR and the SNc, it remains to be tested whether wide field cells would also project to the same Pitx2 modules as narrow field cells, but selectively to MLR/SNc projecting Pitx2^ON^ neurons. In such hypothesised scenario, each Pitx2^ON^ module would contain separate sets of neurons projecting either to the ZI or MLR/SNc, that would be involved in sequential phases of hunting, but that would share the same spatial register with respect to the metric of the necessary orienting movements. SCs: Superficial layer of the Superior Colliculus, SCi: Intermediate and deep layers of the Superior Colliculus. ZI: Zona Incerta. MLR: mesencephalic locomotor region. SNc: substantia nigra pars compacta. Grey arrows indicate predicted connections between cell types based on similar effects on hunting.

Moving into the intermediate layers of the superior colliculus, two overlapping neuronal populations were found to control close range attacks on preys [17,23]. These were glutamatergic neurons projecting to the zona incerta that were also found to strongly overlap with neurons expressing the transcription factor Pitx2 [17,23,27]. Two studies showed that activating both SC ZI-projecting neurons and SC Pitx2^ON^ neurons promoted hunting and even the adoption of a hunter’s posture in the absence of prey [17,23]. However, activating these neurons did not increase the aggression of mice towards conspecifics neither the frequency of food approach [17], arguing that these neurons encode the specific drive to hunt. Also, inactivating these neurons impaired mice’s hunting efficiency. Authors also recorded the firing of SC ZI-projecting neurons while mice captured a prey and found that they rather fired before the initiation of jaw attacks than when mice approached the prey. In addition, these neurons responded to visual or vibrissae stimulation. Taken together, these results show that Pitx2^ON^ and zona incerta projecting neurons in the SC appear to signal the specific drive to attack a prey in close range. It should be noted that Pitx2^ON^ neurons have been shown to project to multiple subcortical targets [23,27] in addition to zona incerta. It is therefore possible, albeit still unproven, that the population of Pitx2^ON^ neurons projecting to the zona incerta is functionally and molecularly separable from the Pitx2^ON^ population projecting along the tectospinal system typically associated with head-eye re-orienting responses. Furthermore, Pitx2^ON^ neurons have been shown to form a spatial register for action [27], with discrete and topographically arranged neuronal modules decoding an ordered motor vector field for re-orienting actions, with each module overseeing a specific portion of egocentric space, forming a spatial motor register [28,29]. It would be therefore interesting to understand whether, within this regular spatial motor register, an additional layer of molecular specialization exists that allows the execution of specific ethologically relevant behaviour, such as hunting, towards defined portions of space, by allowing the selective recruitment of subsets of Pitx2^ON^ neurons projecting to specific subcortical targets within each given module (Figure 2).

Furthermore, given the common involvement in hunting behaviour of Pitx2^ON^ neurons and GRP^ON^ narrow field neurons, it would be interesting to understand whether GRP^ON^ neurons provide any direct or indirect input to Pitx2^ON^ neurons. In particular, GRP^ON^ narrow field neurons have been shown to prefer selective visual features, such as specific motion directions [25]. Like-wise, Pitx2^ON^ neurons are tuned to specific movement vectors. While the topological organization of the direction preferences of GRP^ON^ neurons across the SC has not been mapped, understanding the possible existence of connections between GRP^ON^ and Pitx2^ON^ neurons, and, more importantly, the nature of the resulting visuo-motor alignment, might help to shine new light on the fundamental principles of the sensory-motor integration process (see Figure 2).

In addition to cells tuned to close range attacks on preys, two studies from the Peng Cao lab also reported cells in the intermediate layers of the superior colliculus controlling the initiation of approaches towards preys [17,24]. Compared to the cell types described in the previous paragraph, these are anatomically distinct neurons that project either to the mesencephalic loco-motor region (MLR) or the *substantia nigra pars compacta* (SNc). Neuronal recordings revealed that they fire at the moment of approach initiation and bilateral inactivation of these neurons reduced locomotion speed and the frequency of approaches during predatory hunting [17,24]. However, inactivating SNc projecting neurons did not affect the ability to attack or catch preys nor the frequency of jaw attacks or exploratory and escape speed. This shows that in addition of being anatomically distinct from intermediate layers’ neurons controlling jaw attacks, these neurons control a different phase of the hunting cycle, namely the approach towards the prey. However, activating these neurons also increased appetitive locomotion towards food and conspecifics. Therefore, unlike the ZI-projecting SC neurons, the SC to MLR/SNc pathway seems to have additional roles to those exerted during hunting. Future studies should clarify the different roles and the overlap between neurons projecting to the MLR and the SNc. One aspect to notice is that Pitx2 projection neurons do include projections to both the SNc and the broad MLR area [27], raising the possibility that selective subclasses of Pitx2 neurons might also be involved in the approach phase of the hunting behaviour, which would be in line with their more general role in spatial re-orienting. In addition, given that wide field neurons play a similar role to the MLR and SNc projecting SC neurons, it would be interesting to study the connectivity pattern between these two classes of collicular neurons (Figure 2).

Interestingly, the dichotomy between different circuits involved in different phases of the hunting cycle was even more noticeable in the tectum of fishes [9]. As we mentioned at the end of the perception section, neurons in the rostral part of the tectum prefer larger stimuli than their caudal counterparts, in line with the natural statistics of visual scenes, whereby preys in the binocular zone tend to be close to the fish and those in the monocular zone far from the fish. In turn, this correlates with the metric of the motor neurons in each region, with rostral motor neurons producing small movements suited for prey pursuit and caudal neurons controlling movements of bigger amplitude suited to orient and initiate the approach towards a distant prey [14].

As shown in the previous paragraph and in the first section, different areas of the SC have specific properties that correlate well with the ethological relevant natural statistics of the corresponding portion of the visual field and with the desired motor command. In rodents, this is the case along the medial-lateral axis of the SC. Neurons in the medial SC covering the upper visual field are more sensitive in detecting aerial predator approaching like stimuli and are more likely to produce defensive behaviours such as escaping or freezing [10,16]. In contrast, neurons in the lateral SC overseeing the lower part of the visual field are tuned to detect prey like stimulus and produce appetitive motor commands such as biting [17] and licking [30]. Recent studies dissected the anatomical inputs of neurons along the medial-lateral axis of the mouse SC and revealed the presence of three subdivisions, the medial, lateral and central parts, that receive distinct sets of brain wide inputs [31,32]. This is particularly clear when considering the differential input from the cortex and the *substantia nigra pars reticulata* (SNr). In the case of the SNr, overall the lateral and central parts of the SC receive more input than the medial part. In addition, this input arises from separate group of SNr’s neurons with specific physiological properties. SNr’s neurons projecting to the lateral and central parts of the SC show more rapid action potentials’ rise and fall together with high spontaneous and maximum firing rates, compared with neurons reaching the medial part of the SC that are relatively slower [32]. Regarding the cortical input, different areas project to each of the SC’s medio-lateral subdivisions. For example, the orbitofrontal and primary motor and somatosensory cortices appear to target exclusively the lateral and centro-lateral parts of the SC, whereas the visual and the retrosplenial cortices target mainly the medial and centro-medial parts [31]. Taken together, these results show how different areas of the SC involved in specific behaviours receive specialised input from the brain. Namely, the medial part of the SC receives visuospatial information in line with its role in the detection of aerial predators and the associated escape response towards a shelter location [19], whereas the lateral SC receives somatic and orofacial sensorimotor information in line with its role in prey capture and the associated consummatory phase.

## Cognition. Intrinsic collicular motives supporting cognitive function

As all motor outputs represent the physical endpoint of perceptual and cognitive processes, it should not come as a surprise that recent studies confirmed that, in addition of being a complex reflexive sensorimotor station, collicular circuits play also a critical role in cognitive control. Here, we review some of the most recent studies on the topic.

The main evidence supporting the role of the SC in flexible sensorimotor transformation is the decoupling of sensory and action processing during goal-oriented tasks. SC superficial neurons respond reliably to given visual stimuli in their receptive field [10], and signal salient objects in the visual field even when the animal is engaged in a task [33]. In a classic feedforward sensorimotor model of the SC, the spatial combination of all sensory responses results in the activation of a corresponding selection of motor neurons that produce orienting usually towards the most salient location in the visual field [15]. However, when the animal is engaged in a task, the motor command delivered by the SC is independent of the sensory stimulation [34]. The SC ability to ignore task-irrelevant sensory signals and command movements necessary to achieve the animal’s goal demonstrates its role in supporting adaptive and flexible behaviours based non-only on sensory information but on the internal states and recent experience of the animal.

Nevertheless, despite perceptual and action circuits being able to operate independently, they exert strong influence on each other. Indeed, a recent study in monkeys suggests that except for the outermost dorsal and ventral parts of the superior colliculus with neurons exclusively tuned to vision and movement, respectively, the majority of collicular neurons respond to both sensory and motor signals [35]. This is echoed by three recent studies in mice that showed that approximately a third of neurons in the superficial layers are modulated by movement [36–38]. This shows how action- and perception-related signals are intertwined and often co-encoded by the same collicular neurons.

**Figure 3:**
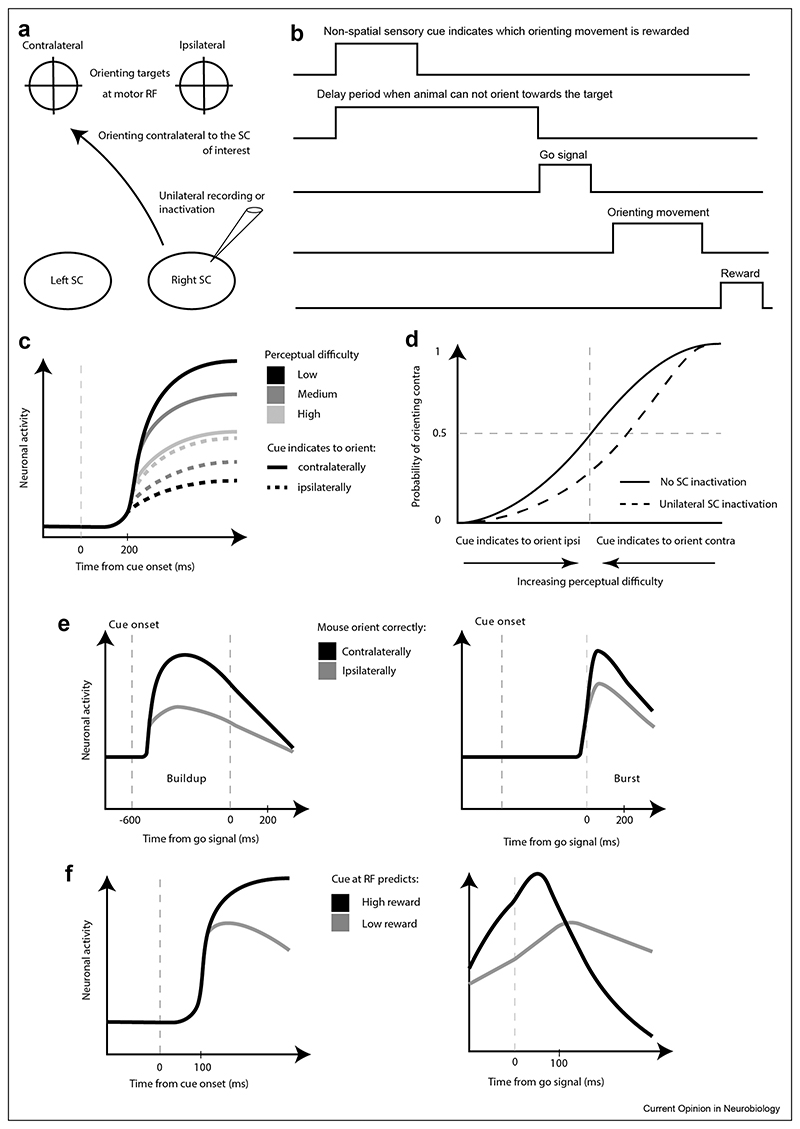
Schematics of a perceptual decision task that has been used in many recent studies to probe the role of the SC in decision making. **a**. Schematics of target spatial locations and illustration of a contralateral orienting movement relative to the superior colliculus studied in one hemisphere. **b**. Schematics of the temporal sequence of events happening during the perceptual decision task. **c**. Representative responses of a neuron in the monkey SC that encodes the perceptual difficulty and the orienting action determined by the stimulus cue [41]. **d**. Representative psychometric curves showing the probability of orienting contralaterally as a function of the perceptual difficulty of the stimulus cue. Unilaterally inactivating the SC creates a bias for orienting ipsilaterally that affects mainly the difficult trials. **e**. Representative neurons in the mouse SC that encode the orienting movement associated with the stimulus cue [39]. On the left is a buildup neuron that exhibit sustained activity and is significantly more active during the delay phase when the mouse correctly orients contralaterally after the go signal. On the right is a burst neuron that fires transiently only after the go signal and significantly more when the mouse correctly orients contralaterally. **f**. Representative responses of a neuron in the monkey SC tuned to the value associated with a visual stimulus [42,43]. Curves on the left correspond to the visual evoked responses following the presentation of two visual stimuli at the same RF location associated with different reward levels. Right is similar to left but for the period around the execution of the orienting movement. RF: receptive field.

Furthermore, several recent studies in mice, rats or monkeys used a similar behaviour task to highlight the role of the superior colliculus in decision making [30,39–41]. Animals were trained to discriminate between two features of a non-spatial sensory cue. For example, mice had to discriminate which odour was more abundant in a mixture of two odours [39] or recognise whether the rate of an auditory click train was below or above 50Hz [30], and monkeys had to discriminate the orientation of a Glass pattern, consisting in two superimposed arrays of dots, where one array is the copy of the other but shifted and rotated, in this case by an angle of either 45 or 135° [41]. Each feature instructed the animal to orient towards one of two targets symmetrically placed on each side of the animal to get a reward (Figure 3a). Two important parameters characterised each trial. First, the perceptual difficulty which corresponded to how discernible were the two stimuli. Second, the delay period occurring after the sensory cue onset, when animals had to refrain from orienting towards a target until a go signal was presented (Figure 3b). These studies revealed that about 200 ms after the cue onset, a sustained increase in the activity of collicular motor neurons could already predict whether the correct choice was going to be performed after the go signal [39,41] (Figure 3c). Interestingly, as the trial difficulty increased, the latency to predict increased and the corresponding level of activity decreased, respectively. These signals could be interpreted as the time it takes to reach a decision and the associated level of confidence. In addition, unilaterally inactivating the SC created an ipsilateral decision bias that affected mainly the success rate of difficult trials [30,39,41] (Figure 3d). Unilaterally inactivating the SC did not affect the execution of the same orienting movement in a different task requiring a low level of perceptual decision [39,41]. Taken together, these results demonstrate that the SC is not only involved in sensory processing, motor preparation and execution of orienting movement, but that it also plays an important role in perceptual decision making. To further support this idea, two recent studies [30,39] used optogenetic approaches to inactivate the SC during specific phases of the decision task. Interestingly, they showed that disrupting the SC during the delay phase caused more orienting mistakes than when inactivating it during the presentation of the sensory cue or after the go signal.

Finally, the learning of a stimulus value can influence the sensory and motor responses of collicular neurons. Authors in [42] presented two spatial targets at the same location to monkeys that were rewarded for orienting towards it. However, one target would predict a bigger reward than the other. They recorded the activity of collicular neurons responding to both the presentation of the targets and the execution of the corresponding orienting movement. They found that neurons exhibited a stronger activation following the presentation of the target predicting a bigger reward (Figure 3f). Similarly, these neurons also fired more when the monkey oriented to get the bigger reward. The increase in motor activity resulted also in faster and early saccades. Therefore, the greater motor activity could underlie an increase in vigour when orienting towards good targets. It is remarkable how the value associated with a stimulus modifies also the sensory encoding in the SC. The greater activity following the presentation of a stimulus associated with a better outcome underlies the SC ability to learn and discriminate between task-relevant stimuli. Similar results were also reported by [43]. This further demonstrates that the SC is not a set saliency detector only reporting and acting on conspicuous or inherent relevant stimuli but can instead adapt its sensory and motor representation to match the goal of the animal.

However, the extent to which the intrinsic organisation of the SC alone is able to support flexible sensorimotor transformations remains to be established. Recent studies have shown that input to the colliculus from the cortex [30] and basal ganglia [40,42,44] play a role in changing the response properties of SC neurons depending on the animal’s state or the environment. In particular in the case of reward value coding, the fact that the response of SC neurons relates to the size of the reward may be a consequence of the direct dopaminergic input provided by the SNr. This input was shown to change the sensibility of SC/tectal neurons to visual stimuli in a way that sensory stimulation becomes more or less effective in triggering a behavioural output [44]. The recent identification of genetic lines giving access to specific neuronal subclasses in the SC in mice as well as novel techniques to map and manipulate neural circuits [45,46] will help to further delineate the collicular networks and dynamics involved in flexible sensorimotor transformations.

## Conclusions

We showed that the study of sensorimotor transformations in the SC is well suited to understand the general principles of how the brain flexibly integrates sensory signals to guide actions. We emphasise that while ethological constraints are likely to have shaped the design of collicular circuits and their computational capabilities, the circuits that have emerged display remarkable capabilities in solving more generalised, non-strictly ethological, problems. Finally, we argued that cognition, perception and action should not be considered as separate entities assignable to separable structures but rather as contiguous, yet potentially separable, dynamical states of the same sensorimotor network. With that regard, the study of the SC appears to provide a tractable and parsimonious system to study and characterise such dynamical states, the circuit motives and molecular features supporting them and the likely psychological functions that such states subtend and to do so across a wide range of species.

## Figures and Tables

**Table 1:** Name and role of specific collicular neurons supporting the different phases of the execution of prey capture.

Name	Function
Narrow field/GRP^ON^	Prey pursuit and attack
Wide field/Ntsr1^ON^	Approach initiation towards the prey from afar
Parvalbumin^ON^	Inactivation does not affect hunting
CLBN2^ON^	Inactivation does not affect hunting
Horizontal cells	Role in hunting not tested
Stellate cells	Role in hunting not tested
Glutamatergic/Pitx2^ON^ neurons projecting to the zona incerta	Drive attack to prey in close range
(Pitx2^ON^?) MLR or SNc projecting neurons	Control the initiation of approaches towards preys

## Data Availability

No data was used for the research described in the article.

## References

[1] Isa T, Marquez-Legorreta E, Grillner S, Scott EK (2021). The tectum/superior colliculus as the vertebrate solution for spatial sensory integration and action. Curr Biol.

[2] Hafed ZM, Chen CY (2016). Sharper, stronger, faster upper visual field representation in primate superior colliculus. Curr Biol.

[3] Li Y, Meister M (2023). Functional cell types in the mouse superior colliculus. Elife.

[4] Kasai M, Isa T (2022). Effects of light isoflurane anesthesia on organization of direction and orientation selectivity in the superficial layer of the mouse superior colliculus. J Neurosci.

[5] Li Y, Turan Z, Meister M (2020). Functional architecture of motion direction in the mouse superior colliculus. Curr Biol.

[6] Feinberg EH, Meister M (2014). Orientation columns in the mouse superior colliculus. Nature.

[7] de Malmazet D, Kühn NK, Farrow K (2018). Retinotopic separation of nasal and temporal motion selectivity in the mouse superior colliculus. Curr Biol.

[8] Ahmadlou M, Heimel JA (2015). Preference for concentric orientations in the mouse superior colliculus. Nat Commun.

[9] Förster D, Helmbrecht TO, Mearns DS, Jordan L, Mokayes N, Baier H (2020). Retinotectal circuitry of larval zebrafish is adapted to detection and pursuit of prey. Elife.

[10] Lee KH, Tran A, Turan Z, Meister M (2020). The sifting of visual information in the superior colliculus. Elife.

[11] Johnson KP, Fitzpatrick MJ, Zhao L, Wang B, McCracken S, Williams PR, Kerschensteiner D (2021). Cell-type-specific binocular vision guides predation in mice. Neuron.

[12] Allen K, Gonzalez-Olvera R, Kumar M, Feng T, Pieraut S, Hoy JL (2022). A binocular perception deficit characterizes prey pursuit in developing mice. iScience.

[13] Chen H, Savier EL, DePiero VJ, Cang J (2021). Lack of evidence for stereotypical direction columns in the mouse superior colliculus. J Neurosci.

[14] Mearns DS, Donovan JC, Fernandes AM, Semmelhack JL, Baier H (2020). Deconstructing hunting behavior reveals a tightly coupled stimulus-response loop. Curr Biol.

[15] Suzuki DG, Pérez-Fernández J, Wibble T, Kardamakis AA, Grillner S (2019). The role of the optic tectum for visually evoked orienting and evasive movements. Proc Natl Acad Sci USA.

[16] Branco T, Redgrave P (2020). The neural basis of escape behavior in vertebrates. Annu Rev Neurosci.

[17] Shang C, Liu A, Li D, Xie Z, Chen Z, Huang M, Li Y, Wang Y, Shen WL, Cao P (2019). A subcortical excitatory circuit for sensory-triggered predatory hunting in mice. Nat Neurosci.

[18] Hoy JL, Bishop HI, Niell CM (2019). Defined cell types in superior colliculus Make distinct Contributions to prey capture behavior in the mouse. Curr Biol.

[19] Campagner D, Vale R, Tan YL, Iordanidou P, Pavón Arocas O, Claudi F, Stempel AV, Keshavarzi S, Petersen RS, Margrie TW (2023). A cortico-collicular circuit for orienting to shelter during escape. Nature.

[20] Wu Q, Li E, Zhang Y (2023). A synaptic filtering mechanism in visual threat identification in mouse. Proc Natl Acad Sci USA.

[21] Montardy Q, Zhou Z, Li L, Yang Q, Lei Z, Feng X, Chen S, Shi Q, Zhang H, Chen S (2022). Dopamine modulates visual threat processing in the superior colliculus via D2 receptors. iScience.

[22] Zhou Z, Liu X, Chen S, Zhang Z, Liu Y, Montardy Q, Tang Y, Wei P, Liu N, Li L (2019). A VTA GABAergic neural circuit mediates visually evoked innate defensive responses. Neuron.

[23] Xie Z, Wang M, Liu Z, Shang C, Zhang C, Sun L, Gu H, Ran G, Pei Q, Ma Q (2021). Transcriptomic encoding of sensorimotor transformation in the midbrain. Elife.

[24] Huang M, Li D, Cheng X, Pei Q, Xie Z, Gu H, Zhang X, Chen Z, Liu A, Wang Y (2021). The tectonigral pathway regulates appetitive locomotion in predatory hunting in mice. Nat Commun.

[25] Gale SD, Murphy GJ (2014). Distinct representation and distribution of visual information by specific cell types in mouse superficial superior colliculus. J Neurosci.

[26] Tsai NY, Wang F, Toma K, Yin C, Takatoh J, Pai EL, Wu K, Matcham AC, Yin L, Dang EJ (2022). Trans-Seq maps a selective mammalian retinotectal synapse instructed by Nephronectin. Nat Neurosci.

[27] Masullo L, Mariotti L, Alexandre N, Freire-Pritchett P, Boulanger J, Tripodi M (2019). Genetically defined functional modules for spatial orienting in the mouse superior colliculus. Curr Biol.

[28] Krauzlis RJ, Lovejoy LP, Zénon A (2013). Superior colliculus and visual spatial attention. Annu Rev Neurosci.

[29] Wilson JJ, Alexandre N, Trentin C, Tripodi M (2018). Three-dimensional representation of motor space in the mouse superior colliculus. Curr Biol.

[30] Duan CA, Pan Y, Ma G, Zhou T, Zhang S, Xu N (2021). A cortico-collicular pathway for motor planning in a memorydependent perceptual decision task. Nat Commun.

[31] Benavidez NL, Bienkowski MS, Zhu M, Garcia LH, Fayzullina M, Gao L, Bowman I, Gou L, Khanjani N, Cotter KR (2021). Organization of the inputs and outputs of the mouse superior colliculus. Nat Commun.

[32] McElvain LE, Chen Y, Moore JD, Brigidi GS, Bloodgood BL, Lim BK, Costa RM, Kleinfeld D (2021). Specific populations of basal ganglia output neurons target distinct brain stem areas while collateralizing throughout the diencephalon. Neuron.

[33] White BJ, Itti L, Munoz DP (2019). Superior colliculus encodes visual saliency during smooth pursuit eye movements. Eur J Neurosci.

[34] McPeek RM, Keller EL (2002). Saccade target selection in the superior colliculus during a visual search task. J Neurophysiol.

[35] Massot C, Jagadisan UK, Gandhi NJ (2019). Sensorimotor transformation elicits systematic patterns of activity along the dorsoventral extent of the superior colliculus in the macaque monkey. Commun Biol.

[36] Schröder S, Steinmetz NA, Krumin M, Pachitariu M, Rizzi M, Lagnado L, Harris KD, Carandini M (2020). Arousal modulates retinal output. Neuron.

[37] Savier EL, Chen H, Cang J (2019). Effects of locomotion on visual responses in the mouse superior colliculus. J Neurosci.

[38] Ito S, Feldheim DA, Litke AM (2017). Segregation of visual response properties in the mouse superior colliculus and their modulation during locomotion. J Neurosci.

[39] Lintz MJ, Essig J, Zylberberg J, Felsen G (2019). Spatial representations in the superior colliculus are modulated by competition among targets. Neuroscience.

[40] Lee J, Sabatini BL (2021). Striatal indirect pathway mediates exploration via collicular competition. Nature.

[41] Jun EJ, Bautista AR, Nunez MD, Allen DC, Tak JH, Alvarez E, Basso MA (2021). Causal role for the primate superior colliculus in the computation of evidence for perceptual decisions. Nat Neurosci.

[42] Amita H, Kim HF, Inoue K, Takada M, Hikosaka O (2020). Optogenetic manipulation of a value-coding pathway from the primate caudate tail facilitates saccadic gaze shift. Nat Commun.

[43] Zhang B, Kan JYY, Yang M, Wang X, Tu J, Dorris MC (2021). Transforming absolute value to categorical choice in primate superior colliculus during value-based decision making. Nat Commun.

[44] Pérez-Fernández J, Kardamakis AA, Suzuki DG, Robertson B, Grillner S (2017). Direct dopaminergic projections from the SNc modulate visuomotor transformation in the lamprey tectum. Neuron.

[45] Lee H, Ciabatti E, González-Rueda A, Williams E, Nugent F, Mookerjee S, Morgese F, Tripodi M (2023). Combining long-term circuit mapping and network transcriptomics with SiR-N2c. Nat Methods.

[46] Ciabatti E, González-Rueda A, Mariotti L, Morgese F, Tripodi M (2017). Life-Long genetic and functional access to neural circuits using self-inactivating rabies virus. Cell.

